# Online and Blended Learning Courses for Healthcare Professionals and Policymakers on Patients’ Perspectives on Medicine: A Project Report

**DOI:** 10.3390/pharmacy10020039

**Published:** 2022-03-16

**Authors:** Ramune Jacobsen, Anna Birna Almarsdóttir, Lourdes Cantarero-Arevalo, Anne Gerd Granås, Johanne M. Hansen, Martin C. Henman, Solveig N. Jacobsen, Susanne Kaae, Lotte S. Nørgaard, Katja Taxis, Sofia K. Sporrong

**Affiliations:** 1 Social and Clinical Pharmacy, Department of Pharmacy, University of Copenhagen, 2100 Copenhagen, Denmark; aba@sund.ku.dk (A.B.A.); lou.cantarero@sund.ku.dk (L.C.-A.); johanne.moelby.hansen@sund.ku.dk (J.M.H.); solveig.nordahl@sund.ku.dk (S.N.J.); susanne.kaae@sund.ku.dk (S.K.); lotte.norgaard@sund.ku.dk (L.S.N.); sofia.sporrong@sund.ku.dk (S.K.S.); 2 Section of Pharmaceuticals and Social Pharmacy, Department of Pharmacy, University of Oslo, 0371 Oslo, Norway; a.g.granas@farmasi.uio.no; 3 Centre for the Practice of Pharmacy, School of Pharmacy and Pharmaceutical Sciences, Trinity College Dublin, D02 PN40 Dublin, Ireland; mhenman@tcd.ie; 4 Pharmacotherapy, Epidemiology and Economics—Groningen Research Institute of Pharmacy, Faculty of Science and Engineering, University of Groningen, 9747 AG Groningen, The Netherlands; k.taxis@rug.nl

**Keywords:** patient perspective, medicine use, massive open online courses, healthcare professional education, qualitative interview

## Abstract

In order for healthcare professionals to better engage with patients, they need to understand and integrate the perspectives of patients into their daily work. In this project, we developed two courses for healthcare professionals on patients’ perspectives on medicine. One course was an online course that introduced the patients’ perspectives on medicine and explained its importance for healthcare and health policy. The second course was a blended learning course, consisting of online modules and face-to-face webinars, which specified how to explore patients’ perspectives in qualitative interviews, and how to develop implementation plans. Patients participated in the development, execution, and evaluation of both courses. Overall, more than 2000 healthcare professionals enrolled in the first course and, in just over a year, 191 participants completed the online course; 57 healthcare professionals registered in the second blended learning course and six participants completed both components of the course. The relevance of knowledge gained was positively evaluated. Participants especially appreciated the participation of patients. Based on the feedback, the second blended learning course was adapted to run online and both courses continue to be freely available to all interested healthcare professionals on the Coursera platform.

## 1. Introduction

For years, there have been many challenges associated with implementing rational use of medications which deserve careful attention [1,2] and now, more than ever, rational use of medications is challenged by the ever-growing use of the Internet and social media [3]. Patients adapt, change, and substitute their treatments often without the involvement of healthcare professionals, based on patient-to-patient interaction and a rapidly increasing amount of information received through the Internet and social media [4]. These situations can, in some cases, cause life-threatening medication errors and waste of resources within healthcare systems [5]. There is also an increasing trend encouraging patients to take more responsibility for their own treatment [6,7,8]. It has been shown that when patients, caregivers, and healthcare professionals jointly decided how to best use medicine, medicine was used more appropriately [9]. However, in order for healthcare professionals and other relevant stakeholders to assess, understand, and involve patients in an effective way, they need appropriate tools and techniques to better acknowledge, understand, and integrate patients’ perspectives on medicine in decisions concerning the medical practices. Therefore, it is important to raise awareness of, and train healthcare professionals and policymakers in, understanding the patients’ perspectives on medicine, and how this could be further strengthened by collecting and analysing qualitative data related to the patients perspectives on medicine.

This paper is a report of the results of a project in which two courses on understanding and researching patients’ perspectives on medicine were designed, developed, delivered, and evaluated. The courses targeted all types of healthcare professionals and health policymakers dealing with patients and medicine. The courses were interactive, i.e., including discussions and sharing participants’ professional experiences. The first course was an online learning course, in which theoretical fundamentals of patients’ perspectives on medicine were discussed, and concrete examples illustrating patients’ challenges with the use of medications were shared. The second course was a blended learning course, in which different phases of qualitative research, i.e., problem definition, qualitative data collection, and data analysis, were presented. These aspects were introduced, explained, exemplified, and tested in interviews with patients. The novelties of the blended learning course, distinguishing it from other existing courses on qualitative research, were: (1) the focus on patients’ perspectives on medicine, (2) the fact that the course was targeted towards healthcare professionals and policymakers, and (3) the option of a blended learning experience, in which course participants met face-to-face (although on-line due to the pandemic) to discuss methodological issues and specific challenges that participants face in practice related to patients’ perspectives on medicine.

## 2. Planning and Development

Initially, 11 academic staff from the Universities of Copenhagen, Groningen, Oslo, Addis Ababa, and Trinity College Dublin, two volunteer patients, and two healthcare professionals attended a kick-off meeting in January 2020. The ideas for the courses were developed in an Arena Blended Connected (ABC) workshop [10], supplemented with Massive Open Online Course (MOOC) requirements provided by Coursera (Mountain View, CA, USA) and adapted by the University of Copenhagen [11].

The format for the ABC workshop was originally developed by the University College London, and then adapted by the Centre of Online and Blended Learning (COBL) at the University of Copenhagen. The workshop lasted 90 min, during which time, two teams (i.e., one team for each of the two courses) created visual “storyboards” that outlined the type and sequence of learning activities required to meet the learning outcomes of the two courses. Six different learning types were presented on different coloured cards (i.e., acquisition, discussion, practice, investigation, collaboration, and production). On each card, conventional and digital learning activities were detailed, for example, activities for acquisition learning included online lectures, face-to-face lectures, reading books and open-source materials, etc. The teams selected the relevant activities for the four online modules in each of the courses and specified the content for each activity. As the workshop progressed, the cards were sequenced on the storyboard sheets, connecting activities, aligning them with learning outcomes, and finally representing the learner journey. A professional from COBL facilitated the workshop and documented its outcomes.

On day two, the academic staff revised the materials created the day before. When consensus was reached, the content of each module was finalised, including writing a manuscript for the lectures, creating quizzes, etc. The contents of modules were structured considering the MOOC requirements for the course outline, including video lectures with quizzes, as well as open-source materials to read or watch (e.g., books and YouTube videos, respectively); discussion prompts; and peer-reviewed and instructor-reviewed assignments. Acquisition learning activities included video lectures and open source materials; discussion learning activites promoted discussion; and peer- and instructor-reviewed assignments covered the practice, investigation, production and collaboration types of learning.

Two academic staff members were assigned to review and scrutinize the finalised content of all the modules to assure a logical flow and appropriate use of terminology. An expert from COBL with a journalist background conducted the final review of the manuscripts for the video lectures, created visual backgrounds, and filmed the lectures. The preparation of the courses lasted from January to September 2020.

## 3. Content

**Figure 1:**
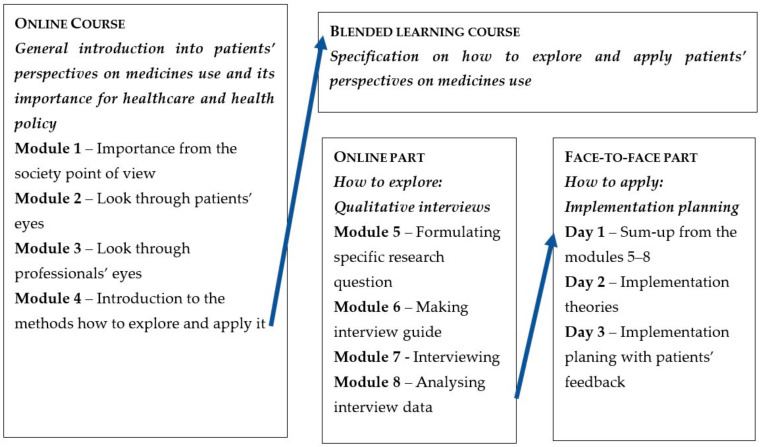
Overall overview and flow of the online and blended learning courses.

An overview of the two courses is presented in Figure 1. The online course (Modules 1–4) was developed as a Massive Open Online Course (MOOC) on the Coursera platform [12]. The blended learning course consisted of: (1) online modules including staff interactions and (2) face-to-face webinars. The blended learning course was provided on the online learning platform Canvas/Absalon (Salt Lake City, UT, USA) at the University of Copenhagen, and participants were required to sign up to get access. The online modules of the blended learning course (Modules 5–8) were held from October to November 2020. The face-to-face webinars were held on three days during one week in November 2020, i.e., Monday, Wednesday, and Friday, from 9 am to 1 pm.

The online course (Modules 1–4) introduced how the patients’ perspectives on medicine could be understood and explained why it was important to explore and apply patients’ perspectives in healthcare practice and in health policy. The online component of the blended learning course (Modules 5–8) specified how qualitative interviews could be used to explore patients’ perspectives on medicine, while the face-to-face webinars focused on the implementation of patients’ perspectives in healthcare and health policy. A detailed description of the aims, learning objectives, learning materials, and assessments of both courses and their components is provided below. The courses were free of charge.

### 3.1. Module 1

**Aim and Learning Objectives**

The first module, entitled “Why is the patient perspective important?”, provided an overview of society-wide challenges related to medicine use. The learning objectives of the module were:

- To explain why it is important to understand patients’ perspectives on medicine from a society’s point of view;
- To provide examples of problems related to medicine use.

**Teaching Materials**

The teaching materials included video lectures, compulsory readings, a discussion prompt, a quiz, and additional readings. The three video lectures lasted, in total, 18 min, and were entitled:

- “Introduction to the course” (1 min);
- “Medicine-related problems from professional and political viewpoints” (10 min);
- “Why we need to understand patients’ perspectives” (7 min).

The compulsory readings were estimated to take, in total, 1 h and 45 min, and dealt with:

- Medicine safety [1];
- Adherence problems [2];
- Shared decision making [4].

Then, the participants had an opportunity to discuss the challenges of medicine use experienced by patients in their daily living, in a discussion prompt, as well as to engage with some additional readings detailing medication safety and adherence problems [13,14,15].

**Participant Assessment**

The module was passed by correctly answering 80% of an eight-question quiz based on the video lectures and compulsory reading materials.

### 3.2. Module 2

**Aims and Learning Objectives**

In the second module, entitled “Patient stories”, the participants learned directly from patients about their views on medicine and how medications are incorporated into patients’ daily living. Furthermore, the module introduced a theoretical model for summarizing different aspects of patients’ perspectives on medicine. The learning objectives of the module were:

- To provide examples of the different factors influencing patients’ experiences with medications in daily living;
- To explain why patients sometimes self-regulate their medications.

**Teaching Materials**

The teaching materials included video lectures, compulsory readings, a discussion prompt, a quiz, and additional readings. The three video lectures lasted, in total, 26 min, and included:

- A lecture presenting and illustrating the Patient Lived Experiences with Medicine (PLEM) model [16] (14 min);
- An interview with an older woman with arthritis, in which the practicalities related to the use of medications are discussed, as well as about the “silent knowledge” a patient often possesses and not always discloses (9 min);
- An interview with a younger woman with multiple chronic diseases, who described her coping strategies and expectations of healthcare professionals (13 min).

Compulsory reading materials included:

- A review article on the PLEM model [16], estimated to be read in 45 min;
- Additional videos with patients taking pain medicines [17], estimated to take about 30 min to watch.

Then, the participants had the opportunity to discuss their views on patients’ self-regulation of their medications, in a discussion prompt, and to read some additional studies, based on qualitative interviews with patients [18,19,20].

**Participant Assessment**

The module was passed by correctly answering 80% of a nine-question quiz based on the video lectures and compulsory reading materials.

### 3.3. Module 3

**Aims and Learning Objectives**

The third module, entitled “The professional perspective”, taught the participants that patients’ rationalities regarding medicine use can be, and often are, different from the rationalities expressed by professionals. The module also introduced various ways that healthcare professionals evaluate patients’ perspectives on medicine, and enabled participants to reflect upon their own professional assumptions about patients’ perspectives on medicine. Specific learning objectives of the module were:

- To provide examples of how healthcare professionals and policymakers view and detect patients’ perspectives on medicine, using questionnaires on (a) satisfaction, (b) patients’ attitudes towards medicine, and (c) knowledge of, or behaviours related to, medications;
- To reflect on how the assumptions and perceptions of the participants concerning patients’ perceptions on medicine are different or similar to those of patients.

**Teaching materials**

The teaching materials consisted of video lectures, compulsory readings, a discussion prompt, a quiz, and additional readings. The three video lectures lasted, in total, 19 min, and were entitled:

- “The professional vs. the patient perspective” (3 min);
- “Interactions between patients and professionals” (8 min);
- “Evaluation methods used by professionals” (8 min).

Compulsory readings were estimated to take, in total, 1 h and 40 min and included:

- A report on different aspects of the concept “patient perspective” provided in the literature, prepared by the academic staff from the University of Copenhagen [21];
- Readings on medication adherence measures [22];
- An example of the medication burden questionnaire [23].

Then, the participants had an opportunity to discuss their own perspectives on medications as compared with those of patients (in a discussion prompt), and to read more about questionnaires used by healthcare professionals to assess different aspects of medical treatment (in additional readings).

**Participant Assessment**

The module was passed by correctly answering 80% of a nine-question quiz based on video lectures and compulsory reading materials.

### 3.4. Module 4

**Aims and Learning Objectives**

The fourth and final module of the online course was entitled “How to apply and explore patients’ perspectives”. In this module, the participants were shown examples of how patients’ perspectives have been implemented into health policy and healthcare practice, and participants were acquainted with different aspects of a qualitative interview as a method that could be used for an in-depth exploration of patients’ perspectives. Lastly, the participants reflected on how they could use the knowledge obtained from the course in their professional settings. The learning objectives of this module were:

- To provide examples of how to apply patients’ perspectives on medicine in professional settings;
- To provide examples of the methods that can be used for in-depth exploration of a patient’s perspective on medicine;
- To explain the ethical aspects when exploring patients’ perspectives on medicine.

**Teaching Materials**

The teaching materials included video lectures, compulsory readings, discussion prompts, additional readings, and a written assignment. The four video lectures lasted, in total, 32 min. The contents of the lectures are described below:

- The first lecture provided examples of how patients’ perspectives on medicine can be applied in a healthcare setting (5 min);
- The second lecture was an interview with a patient representative who described their experiences of being involved as a patient in different professional settings related to the development and use of medications (16 min);
- The third lecture was devoted to qualitative methods and provided an overview of these methods (6 min);
- The fourth lecture was also devoted to qualitative methods and presented related ethical principles (5 min).

The compulsory readings in this module consisted of literature on how to conduct qualitative research [24] and was estimated to take 30 min to read.

Then, the participants discussed what they thought about the methods, and what they had learned during the course in two discussion prompts. They could also watch additional videos related to patient involvement, as one of the ways to apply patients’ perspectives [25].

**Participant Assessment**

The participants had to submit a written assignment, describing how patients’ perspectives were currently applied in their professional setting, and how this situation might be improved. After submitting their own assignment, the participants had to evaluate the assignments of at least two other participants. The module and the online course were passed if the peer evaluators gave at least eight out of maximum ten points, for the written assignment.

### 3.5. Module 5

**Aim and Learning Objectives**

The module entitled “How do we design a qualitative interview study?” started with a presentation of the different stages of a qualitative interview study. Then, insight into the different relevant themes that could be explored via interviews was introduced. Finally, the participants designed a small qualitative interview study to be carried out throughout the remaining modules of the online component of the blended learning course, in which each module covered a separate phase in the process. The learning objectives of this module were:

- To understand the relevance of the different stages of a qualitative interview study;
- To define a research question within the area of patients’ perspectives on medicine, which was relevant to a participant’s own professional setting;
- To design a small qualitative interview study to answer this research question.

**Teaching Materials**

The teaching materials included video lectures, a quiz, a discussion prompt, and an instructor-reviewed assignment. The five video lectures lasted, in total, 1 h and presented the following:

- The theoretical basis of a qualitative interview study (i.e., an outline and short presentation of the seven phases of a qualitative interview study);
- An interview with a researcher on how and why interview studies can be carried out;
- An interview with a pharmacist who described their experience carrying out an interview study in a pharmacy;
- An interview with a policymaker about what kind of interview studies with patients they had found relevant from a health policy point of view;
- An interview with a patient who explained his reasons for recommending interview studies to be carried out.

After the theoretical part, the participants had to answer at least 80% of a seven-question quiz. Then, they discussed their research questions with peers in a discussion prompt and looked at some additional interview studies as additional readings [26,27].

**Participant Assessment**

The module was passed by submitting the research question to be worked with during the course, along with the design of a relevant interview study for the course instructors to provide feedback.

### 3.6. Module 6

**Aims and Learning Objectives**

This module, entitled “Preparing for an interview”, aimed at supporting the participants in identifying relevant materials to prepare an interview guide. The module illustrated other central elements to consider when creating the interview guide. In addition, ethical considerations, strategies, and practical advice for recruiting participants were covered. The specific learning objectives of the module were:

- To create an interview guide;
- To explain different recruitment strategies and to select the most suitable strategy for participants’ own studies.

**Teaching Materials**

The teaching materials included video lectures, additional readings, a discussion prompt, compulsory readings, and an instructor-reviewed assignment. The five video lectures lasted, in total, 36 min. The content of the lectures covered:

- Preparation for an interview study (13 min);
- Construction of the interview guide (7 min);
- Informed consent (3 min);
- Recruitment (7 min);
- Sampling strategies for an interview study (6 min).

Additionally, the participants were provided with a YouTube video which provided tips on how to design a semi-structured interview guide [28]. They could also discuss their interview guides with peers in a discussion prompt. Compulsory reading materials consisted of papers relevant to individual participants’ interview studies, selected by the course instructors, estimated to be read in a maximum of 2 h.

**Participant Assessment**

To pass the module, the participants had to submit their interview guide and informed consent form for the course instructors to provide feedback.

### 3.7. Module 7

**Aims and Learning Objectives**

This module was entitled “How to conduct an interview”. During the week allocated to this module, the participants had to conduct at least one interview to understand patients’ perspectives on medicine, using the guide from the previous module. Additionally, the module supported the participants in developing their skills as interviewers, emphasizing body language, empathy, and active listening. This module also introduced the most common pitfalls in interview situations and provided time for reflections on participants’ own interviewing skills. The specific learning objectives of the module were:

- To explain the do’s and don’ts of conducting interviews;
- To conduct a qualitative interview;
- To discuss the participants’ own roles as interviewers.

**Teaching Materials**

The teaching materials included a video lecture, a quiz, additional readings, a discussion prompt, and an instructor-reviewed assignment. The video lecture lasted 12 min and was devoted to interview preparation, phases, and skills. The lecture was followed by a two-question quiz. Further, the participants were provided with additional literature on interviewing [29,30], and they could discuss their interviewing skills with peers in a discussion prompt.

**Participant Assessment**

To pass the module, the participants had to conduct at least one interview, with an estimated 2 h for preparation (e.g., arrangement) and execution of this interview.

### 3.8. Module 8

**Aims and Learning Objectives**

This module was entitled “Analysing a qualitative interview” and provided participants with an understanding on how to analyse qualitative interview data. The participants learned the most important points to consider when transcribing interviews and they were provided with an overview of different types of commonly used qualitative analyses. Then, the participants practised their skills by transcribing and analysing parts of their own interview. Finally, they reflected on their experiences throughout Modules 5 to 8. The specific learning objectives of this module were:

- To transcribe an interview;
- To describe approaches when analysing qualitative interview data;
- To discuss experiences with interviewing a patient and with analysing and interpreting the interview findings.

**Teaching Materials**

The teaching materials consisted of two video lectures, quizzes, compulsory readings, a peer-reviewed assignment, and a discussion prompt. The two video lectures lasted, in total, 12 min, and were entitled:

- “The transcription process” (5 min);
- “Data analysis and coding” (7 min).

The lectures were both augmented with three- and four-question quizzes, respectively.

Compulsory reading was estimated to take 1 h and included the analysis part from the qualitative interview guide, prepared by the University of Copenhagen academic staff [31].

**Participant Assessment**

To pass the module and the online part of the blended learning course, the participants had to submit an assignment in which they transcribed and analysed their own interview data, spending a maximum of 2 h on the job. Further, they had to evaluate the assignments of at least two peers. Finally, to prepare for the face-to-face webinars of the blended learning course, in a discussion prompt, they had to discuss their experiences throughout the course associated with conducting an interview concerning a patient’s perspective on medicine.

### 3.9. Face-to-Face Webinars

**Aim and Learning Objectives**

The overall aim of the face-to-face webinars (which, before the COVID-19 pandemic, were planned to be carried out as physical workshops at the University of Copenhagen) was to teach the participants how to apply patients’ perspectives to develop services contributing to better use of medications. The learning objectives of the face-to-face webinars were:

- To discuss and clarify questions and challenges related to participants’ interview studies concerning patients’ perspectives on medicine, which were conducted during Modules 5–8;
- To become acquainted with implementation strategies;
- To develop an implementation plan for how to incorporate the learnings from the interview study conducted during Modules 5–8, into daily practice.

**Teaching Content**

The teaching content of each day is specified below.

Day 1 was allocated to introduce each other’s projects (1.5 h) and to discuss the challenges while conducting individual interviews (1.5 h). During the last hour, the subject of implementation and the readings to prepare for the next session were introduced by the course instructors. The readings included:

- A paper on Consolidated Framework for Implementation Research (CFIR) [32];
- A paper on Systems Engineering Initiative for Patient Safety (SEIPS) [33].

Day 2 started with the lectures on implementation strategies (1 h and 15 min), where the participants were provided with a template to be used for making an implementation plan. Then, the participants worked individually to develop their individual implementation plans, which were then shared and discussed in groups with peers and course instructors (1 h and 45 min). During the next day free of teaching, the participants had to finalise their individual implementation plans.

Day 3 was the last day of face-to-face webinars and the blended learning course, and it started with group work, where the finalised individual implementation plans were presented, and commented upon by peers, course instructors, and patients (3 h). The last hour of the day, and of the entire blended learning course, was devoted to course evaluation.

## 4. Evaluation

### 4.1. Online Course: Modules 1–4

**Evaluation Aims**

The aims of the evaluation of the online course were: (1) to assess the activity levels in the course, (2) to describe the demographics of the participants completing the course, and (3) to assess how the participants evaluated the course in terms of teaching methods and content.

**Evaluation Methods**

The activity levels were assessed based on the information provided by the Coursera statistics. The demography of participants and their evaluations of the teaching methods and content were based on a questionnaire, including open-ended questions (Appendix A), constructed by the University of Copenhagen academic staff and delivered using the SurveyXact online survey system (Aarhus, Denmark) to the participants completing the course.

**Evaluation Results**

According to Coursera statistics, starting from the end of September 2020 until the middle of December 2021 (i.e., for slightly more than a year), the course page had 10,405 unique visitors, of which 2249 people enrolled into the course. Among the people who enrolled, 1053 were participants who started the course, of which 191 participants completed the course, and 90 of the participants who completed the course responded to at least one question in the evaluation questionnaire. Among the respondents, the majority were (a) women, (b) employed in the health care field, (c) who had worked in their field up to 10 years. The median age of the participants was 30 years old, and they came from four continents around the world, mostly from Europe and Asia (Table 1).

The results of the evaluation of the online course were excellent. The relevance of the knowledge provided in the course was rated the highest. Among the course elements, the videos received the best evaluations. When evaluating the individual modules, Module 2 received the best evaluation; almost all participants “completely agreed” that they “got insight into different factors influencing patients’ experiences with medicine in daily living” (Table 2).

Accordingly, the answers to the open-ended questions revealed that the participants considered the course to be “extremely interesting”, “inspiring”, “excellent”, “relevant” and “engaging”. They especially liked the presentations of the patient stories, and the variety of teaching materials, i.e., the combination of videos, readings, quizzes, discussions, and assignments. They also appreciated the opportunity “to write to the peers” and put their “reflections and reality there”. Regarding possible improvements, three main issues were mentioned: a need for a more specific introduction to the course; some replacement of workload from Module 4 to Module 3; and a suggestion for the discussions to be mandatory.

### 4.2. Blended Learning Course

**Evaluation Aims**

Similar to the aims of the online course evaluation, the aims of the evaluation of the blended learning course were: (1) to assess activity levels in the course; (2) to describe demographic characteristics of the course participants; and (3) to assess how the participants evaluate the course in terms of teaching methods and content.

**Evaluation Methods**

The activity levels in the course were assessed based on the number of people registering (by e-mail) for the course, and the number of people completing the course assignments using the University of Copenhagen teaching platform. The participants’ demographics and their evaluations of the content and teaching methods in the online modules of the blended learning course (Modules 5–8) were based on a survey provided by EIT Health (i.e., the funder) and adapted by the University of Copenhagen academic staff. Similar to the survey used to evaluate Modules 1–4 of the online course, the survey for the Modules 5–8 of the blended learning course included both closed and open-ended questions. The evaluation of the content and teaching methods of the face-to-face webinars of the blended learning course were based on discussions with the course participants during the last hour of the face-to-face webinars. The contents of these discussions were analysed qualitatively.

**Evaluation Results**

In total, 57 persons registered for the blended learning course, among whom, 19 people logged in to access the materials for the online modules (Modules 5–8). Seven people completed all the assignments here, among whom, six people participated in the face-to-face webinars. One person dropped out from the webinars due to personal reasons. Among the six participants, one was male and five were female. Three participants were aged 35–44 years, two were younger, and one was older. Three participants were from Europe, two participants were from Africa, and one participant was from Asia. One participant was a postgraduate student, and the other five participants were pharmacists employed in academia, administration or pharmacies.

Overall, the evaluation of the online modules of the blended learning courses were positive: half of the participants were “extremely satisfied” with the content of the course, two thirds of the participants were “extremely satisfied” with other participants, and all participants were “extremely satisfied” with the level of the course instructors, as well as the usefulness of the program. While answering the open-ended question “What did you like the most?”, participants noted teaching approaches (e.g., variability, content of the videos, interaction with the teachers) and usability or practical aspects of the course where patients were involved in the teaching. One of the participants wrote, “Putting the learning into practice really helped me to understand the topic better. Also including real patients was very good”.

While answering the open-ended question “What did you like the least?”, the participants mentioned work load (i.e., it seemed to be too heavy for a short period of time for participants with presumably full-time jobs) and the administration of the course material (the materials for the forthcoming week were only opened in the beginning of this week and participants would have liked to have an overview of all the materials at once).

One of the participants wrote, “It was not always clear what work was involved for the next week. That made planning for myself in one week challenging (because of holidays). Maybe for the next time posting a schedule for the whole course might have helped.”

A content analysis of the discussion after the face-to-face webinars revealed four main themes:

- **General feedback** Participants thought that this was an interesting course, with strong content. The course for some participants exceeded their expectations. Participants thought that the level of complexity was right, and the knowledge gained was helpful to understand and apply the patients’ perceptions.
- **Structure** Participants liked being introduced to each other, which made the teaching more personal as compared with the online courses. Some thought that the face-to-face webinars could have been placed earlier in the course.
- **Content** Participants appreciated having patients involved giving feedback and adding new insights to their implementation plans. Some participants would have liked to have had more references to scientific materials, including books.
- **Administration** The group size of six students was thought to be very fitting/appropriate. The participants noted that all the administrative questions were quickly solved. A few participants said that they would have liked to have the readings sent by e-mail ahead of the course.

## 5. Reflections and Perspectives

We developed, implemented, and evaluated two innovative online (Modules 1–4) and blended learning (Module 5–8 and 3-day face-to-face webinars) courses for healthcare professionals and policymakers, to raise awareness of the importance of patients’ perspectives on medicine and to teach qualitative interviews as a method to research and to understand patients’ perspectives on medicine. The attendance and evaluations of the online course to introduce patients’ perspectives on medicine were excellent. Despite good evaluations, participants’ attendance in the blended learning course’s teaching interview methods was low. This may be related to the high intensity of the course and the need to be present for the teaching at certain hours of the day during the three-day face-to-face webinars.

We plan to continue working on attracting a broader audience of healthcare professionals and policymakers to the courses, and thereby, create awareness of the importance of patients’ perspectives on medicine for healthcare practice and health policy. The decisions on how to reach this goal were taken during academic staff discussions in the final evaluation meeting, taking into consideration the suggestions of the evaluation panel, including external experts in designing educational activities for healthcare professionals and a patient representative.

In order to reach a broader audience for the online course (Modules 1–4), it was decided, firstly, to intensify the marketing of the course, to try to reach the USA, and to make a promotional video [34], and secondly, to use the course for teaching internal and external university students. The latter decision has already been implemented, and the course is used for teaching pharmacy students at the University of Copenhagen and the University of Oslo. In order to reach a broader audience for the online modules of the blended learning course (Modules 5–8), it was decided to transfer the course to a more user-friendly educational platform, i.e., Coursera, after replacing instructor reviews with peer reviews and quizzes, which the University of Copenhagen academic staff took responsibility for. The course is already running on Coursera [35] and it is being marketed with a promotional video [36]. Similar to Modules 1–4, Modules 5–8 are also being used for teaching both our own and external university students (i.e., the videos from the course were recently used in a summer school by the European Drug Utilisation Research Group (EuroDURG)). We decided to not continue providing the face-to-face webinars, due to the very small number of participants and the heavy workload for the instructors.

## 6. Conclusions

In the two courses on patients’ perspectives on medicine and how these perspectives can be explored in qualitative interviews, healthcare professionals learned to appreciate patients’ perspectives on medicine. This awareness is important, so that medications can be tailored to individual patient needs, potentially reducing medication harm and leading to improvements in health outcomes. Currently, the courses are available on the Coursera platform, at no cost, to all interested healthcare professionals, filling a gap in the existing Coursera portfolio of courses for healthcare professionals. The courses have, so far, received positive feedback from participants from several continents.

## Figures and Tables

**Table 1:** Characteristics of the participants of the online course (Modules 1–4), who completed the course and responded to the evaluation survey.

Characteristics		N (%) Unless Other Indicated
Gender (N = 76)		
	Women	51 (67%)
	Men	25 (33%)
Age (N = 62)		
	Years, Mean (SD)	33.7 (12.37)
	Years, Median (IQR)	30 (24–43)
Field of work (N = 72)		
	Health care	37 (52%)
	Health policy	2 (3%)
	Pharmaceutical industry	10 (14%)
	Academia	13 (18%)
	Other (e.g., students)	10 (14%)
Years of experience in this field (N = 73)		
	Less than 1 year	15 (21%)
	1 to 10 years	39 (53%)
	More than 10 years	19 (26%)
Continent (N = 75)		
	Europe	33 (44%)
	Asia	31 (41%)
	Africa	7 (9%)
	South America	3 (4%)
	North America	2 (3%)

**Table 2:** Results of the survey evaluating the online course (Modules 1–4).

	On a Scale from 1 to 5, i.e., from 1, Not at All, to 5, Completely				
	Mean	SD	95% CI	Median	IQR
**General evaluation**					
The knowledge I gained was relevant to me (N = 90)	4.6	0.79	4.4–4.8	5	4–5
The course improved my knowledge about patients’ perspectives on the use of medications (N = 90)	4.5	0.85	4.3–4.7	5	4–5
The videos were interesting (N = 89)	4.5	0.91	4.3–4.7	5	4–5
The course had a proper level of detail (N = 88)	4.4	0.85	4.2–4.6	5	4–5
The quizzes helped me remember the matters presented (N = 90)	4.4	0.90	4.2–4.6	5	4–5
The discussion helped reflecting the matters presented (N = 89)	4.3	0.88	4.1–4.5	5	4–5
The reading materials were interesting (N = 89)	4.3	0.97	4.1–4.5	5	4–5
The course had an appropriate workload (N = 90)	4.3	0.97	4.1–4.5	5	4–5
**Meeting objectives of Module 1**					
Explain why it is important to understand patients’ perspectives on medication use related to these society-wide challenges (N = 78)	4.7	0.49	4.6–4.8	5	4–5
Give examples of medication use related problems at the societal level (N = 78)	4.6	0.52	4.5–4.7	5	4–5
**Meeting objectives of Module 2**					
Have insight into different factors influencing patients’ experiences with medications in daily living (N = 76)	4.8	0.47	4.7–4.9	5	5–5
Be able to explain why patients sometimes self-regulate their medications (N = 78)	4.7	0.49	4.6–4.8	5	4–5
**Meeting objectives of Module 3**					
Give examples of how healthcare professionals and policymakers view and register patients’ perspectives on medications (N = 76)	4.6	0.51	4.5–4.7	5	4–5
Explain your own assumptions and perceptions of patients’ use of medications (N = 76)	4.6	0.59	4.5–4.7	5	4–5
**Meeting objectives of Module 4**					
Be aware of ethical aspects when exploring patients’ perspectives on medicine (N = 73)	4.7	0.56	4.6–4.6	5	4–5
Be acquainted with how to apply patients’ perspectives on medicine in professional settings (N = 75)	4.6	0.53	4.5–4.7	5	5–5
Be acquainted with the methods that can be used for in-depth explorations of patients’ perspectives on medicine (N = 76)	4.6	0.51	4.5–4.7	5	4–5

## Data Availability

Fully anonymized data supporting reported results can be found on the password-protected drive of the University of Copenhagen. R.J. takes full responsibility for data safety and accuracy.

## Appendix A. Questionnaire to Evaluate the Online Training on “Fundamentals of Patients’ Perspectives on Medicine Use”

Please answer the questions below to help us to address your training needs better and improve the course.

We will ask you to rate different aspects of the course, as well as to freely express your thoughts in the comments. The latter is especially important as the course is running for the first time, and we value all the feedback, both positive and negative. Any suggestions for improvements are very welcome.

All the responses are completely anonymous.

First, we will ask you about the course in general.

On a scale from 1 to 5, please assess the following:

	**1** **Not at All**	**2**	**3**	**4**	**5** **Completely**
The course improved my knowledge about patients’ perspectives on medicine use					
The knowledge I gained was relevant to me					
The course had a proper workload					
The course had a proper level of detail					
The videos were interested					
The quizzes helped remembering the matters presented					
The discussion helped reflecting the matters presented					
The reading material were interesting					

Free text comments about the course generally: Please write what you think was good and bad, what you would suggest improving

_________________

Now, we will ask you about the specific modules of the course.

To refresh your memory, please visit the description of the modules clicking the link below.

The content of the modules can be found under “Syllabus.”

Please note that the link will open in a separate window.

https://coursera.org/learn/patient-perspectives-on-medications

Module 1.

On a scale from 1 to 5, please assess the if the learning objectives of Module 1 were met.

	**1** **Not at All**	**2**	**3**	**4**	**5** **Completely**
(1) Give examples of medicines use related problems at the societal level.					
(2) Explain why it is important to understand patients’ perspectives on medicines use related to these society-wide challenges.					

Free text comments to Module 1: Please write what you think was good and bad, what you would suggest improving

_________________

Module 2.

On a scale from 1 to 5, please assess the if the learning objectives of the module 2 were met.

	**1** **Not at All**	**2**	**3**	**4**	**5** **Completely**
(1) Have insight into different factors influencing patients’ experiences with medicine in daily living.					
(2) Be able to explain why patients sometimes self-regulate their use of medications.					

Free text comments to Module 2: Please write what you think was good and bad, what you would suggest improving

_________________

Module 3.

On a scale from 1 to 5, please assess the if the learning objectives of the module 3 were met.

	**1** **Not at All**	**2**	**3**	**4**	**5** **Completely**
(1) Give examples of how healthcare professionals and policymakers view and register patients’ perspectives on use of medications.					
(2) Explain your own assumptions and perceptions of patients’ perceptions on the use of medications.					

Free text comments to Module 3: Please write what you think was good and bad, what you would suggest improving.

_________________

Module 4.

On a scale from 1 to 5, please assess the if the learning objectives of Module 3 were met.

	**1** **Not at All**	**2**	**3**	**4**	**5** **Completely**
(1) Be acquainted with how to apply patients’ perspectives on medicine in professional settings.					
(2) Be acquainted with the methods that can be used for an in-depth exploration of patients’ perspectives on medicine.					
(3) Be aware of ethical aspects when exploring patients’ perspectives on the use medicine.					

Free text comments to Module 4: Please write what you think was good and bad, what you would suggest improving.

_________________

Finally, we would like to ask a few questions about you.

You are:

- Female
- Male
- Rather not say

How old are you?

_________________

Which field do you work in?

- Healthcare
- Health policy
- Pharmaceutical industry
- Academia
- Other. Please specify_________________

How long have you been working in this field?

________________

Which country do you live in?

_______________

Thank you for your assistance!
